# Dynamic arterial elastance to predict arterial pressure response to volume loading in preload-dependent patients

**DOI:** 10.1186/cc9420

**Published:** 2011-01-12

**Authors:** Manuel Ignacio Monge García, Anselmo Gil Cano, Manuel Gracia Romero

**Affiliations:** 1 Servicio de Cuidados Críticos y Urgencias, Unidad de Investigación Experimental, Hospital del SAS de Jerez, C/Circunvalación s/n, 11407 Jerez de la Frontera, Spain

## Abstract

**Introduction:**

Hemodynamic resuscitation should be aimed at achieving not only adequate cardiac output but also sufficient mean arterial pressure (MAP) to guarantee adequate tissue perfusion pressure. Since the arterial pressure response to volume expansion (VE) depends on arterial tone, knowing whether a patient is preload-dependent provides only a partial solution to the problem. The objective of this study was to assess the ability of a functional evaluation of arterial tone by dynamic arterial elastance (Ea_dyn_), defined as the pulse pressure variation (PPV) to stroke volume variation (SVV) ratio, to predict the hemodynamic response in MAP to fluid administration in hypotensive, preload-dependent patients with acute circulatory failure.

**Methods:**

We performed a prospective clinical study in an adult medical/surgical intensive care unit in a tertiary care teaching hospital, including 25 patients with controlled mechanical ventilation who were monitored with the Vigileo^® ^monitor, for whom the decision to give fluids was made because of the presence of acute circulatory failure, including arterial hypotension (MAP ≤65 mmHg or systolic arterial pressure <90 mmHg) and preserved preload responsiveness condition, defined as a SVV value ≥10%.

**Results:**

Before fluid infusion, Ea_dyn _was significantly different between MAP responders (MAP increase ≥15% after VE) and MAP nonresponders. VE-induced increases in MAP were strongly correlated with baseline Ea_dyn _(*r*^2 ^= 0.83; *P *< 0.0001). The only predictor of MAP increase was Ea_dyn _(area under the curve, 0.986 ± 0.02; 95% confidence interval (CI), 0.84-1). A baseline Ea_dyn _value >0.89 predicted a MAP increase after fluid administration with a sensitivity of 93.75% (95% CI, 69.8%-99.8%) and a specificity of 100% (95% CI, 66.4%-100%).

**Conclusions:**

Functional assessment of arterial tone by Ea_dyn_, measured as the PVV to SVV ratio, predicted arterial pressure response after volume loading in hypotensive, preload-dependent patients under controlled mechanical ventilation.

## Introduction

Arterial hypotension is always a clinical emergency. A sustained decline in arterial pressure, whatever the mechanism that produced it, leads to a decrease in tissue perfusion pressure, organ dysfunction and finally death. Although fluid administration remains the first-choice therapy, the assumption that increasing stroke volume (SV) arterial pressure will also rise is not always true, since the pressure-volume relationship is not easily predictable and depends on the arterial tone. Thus, for the same increase in SV, the increase in arterial pressure will be greater if the arterial tone is higher [1].

Although systemic vascular resistance (SVR) remains the most common parameter used by clinicians to describe arterial tone, its value only represents the opposition to a mean and constant flow, as it exists mainly at the level of arterioles, where the compensatory mechanisms that control vasomotor tone regulate perfusion pressure within the physiological range [2,3]. However, because of the oscillatory nature of arterial pressure and blood flow, this approximation provides not a full characterization of the whole arterial impedance but just a gross oversimplification, ignoring other components such as arterial compliance, characteristic impedance or arterial wave propagation-reflection phenomena [2,3].

On the basis of the Windkessel model, the arterial pressure could be described as the result of the interaction between left ventricular SV and the arterial system [4-6]. So, the ability of an arterial vessel to increase pressure with increases in flow is related to arterial stiffness and is a function of the slope of the arterial volume-pressure relationship or arterial elastance (Ea), which could be defined as the ratio of changes in pressure to changes in volume. Arterial elastance therefore could be considered an integrative parameter of overall arterial system behavior [3,7].

Recently, Pinsky has advocated the assessment of arterial tone in a dynamic fashion by using cyclic changes in pulse pressure and SV during mechanical ventilation [1,8]. He proposed that measuring the ratio of pulse pressure variation (PPV) to stroke volume variation (SVV) during a single positive-pressure breath could provide a functional evaluation of arterial tone. He argues that the functional assessment by dynamic arterial elastance would allow a continuous and immediate estimation of arterial tone at the bedside and could help to predict which patients will show increased arterial pressure with fluid administration [1,8].

Since the aim of the cardiovascular system is to maintain not only blood flow but also adequate perfusion pressure [9], even if a patient is preload-responsive, knowledge of arterial tone is also an important factor in deciding on the appropriate treatment. The purpose of the present study, therefore, was to assess whether dynamic arterial elastance (Ea_dyn_), defined as the PPV to SVV ratio, could predict the arterial pressure response after volume loading in hypotensive, preload-dependent patients.

## Materials and methods

This study was performed in the 17-bed multidisciplinary Intensive Care Unit of the Hospital de SAS Jerez de la Frontera. The protocol was approved by the Institutional Ethics Committee of the Jerez Hospital of the Andalusian Health Service, and the study was endorsed by the Scientific Committee of the Spanish Society of Intensive Care, Critical and Coronary Units (SEMICYUC). Written informed consent was obtained from each patient's next of kin.

### Patients

The inclusion criteria were patients on controlled mechanical ventilation equipped with an indwelling radial artery catheter connected to the FloTrac/Vigileo hemodynamic monitoring system and for whom the decision to give fluids was made because of the presence of one or more clinical signs of acute circulatory failure, including arterial hypotension (mean arterial pressure (MAP) ≤65 mmHg, systolic arterial pressure (SAP) <90 mmHg or a decrease of 40 mmHg from baseline [10]) and preserved preload dependence condition, defined as the presence of a stable value of SVV ≥10% [11]. Contraindications for the volume administration were based on the evidence of fluid overload and/or of hydrostatic pulmonary edema. Patients with unstable cardiac rhythm were excluded.

### Arterial pulse pressure variation calculation

The arterial pressure waveform was recorded online on a laptop computer at a sampling rate of 300 Hz using proprietary data acquisition software (S/5 Collect software, version 4.0; Datex-Ohmeda, Helsinki, Finland) and converted to ASCII files for *post hoc *offline analysis (QtiPlot software, version 0.9.7.13; ProIndep Serv, Craiova, Romania).

Arterial PPV was defined according to the following known formula:

$$\text{PPV}\left(\%\right)=\text{1}00\times \left({\text{PP}}_{\text{max}}-{\text{PP}}_{\text{min}}\right)/\left[\left({\text{PP}}_{\text{max}}+{\text{PP}}_{\text{min}}\right)/\text{2}\right)],$$

where PP_max _and PP_min _are the maximum and minimum pulse pressures determined during a single respiratory cycle [12]. In order to obtain a consistent PPV value, the average of five consecutive measurements was used for statistical analysis [13].

### Cardiac output and stroke volume variation measurements

A high-fidelity dedicated pressure transducer (FloTrac sensor; Edwards Lifesciences LLC, Irvine, CA, USA) was connected to the arterial line and attached to the Vigileo monitor, software version 3.01 (Edwards Lifesciences LLC). Cardiac output (CO) was calculated on the basis of the real-time analysis of the arterial waveform over a period of 20 seconds. This calculation was performed at a sample rate of 100 Hz without the need for prior calibration using a proprietary algorithm based on the principle that aortic pulse pressure is proportional to SV. SV was measured as the standard deviation (SD) of the arterial pressure around MAP and was inversely related to arterial compliance. The effects of arterial compliance and vascular resistance were estimated every minute on the basis of individual patient demographic data (age, gender, body weight and height) and the arterial waveform shape analysis, respectively, and they were integrated by using a conversion factor known as χ. SVV was assessed every 20 seconds by the system as follows:

$$\text{SVV}\left(\%\right)=\text{1}00\times \left[\left({\text{SV}}_{\text{max}}-{\text{SV}}_{\text{min}}\right)/{\text{SV}}_{\text{mean}}\right].$$

Since SVV was computed over a period of 20 seconds while χ was updated only every minute, the χ factor was constant from one heartbeat to another and hence was eliminated from the equation when calculating SVV as follows [14]:

$$\begin{array}{c} \text{SVV}\left(\%\right)=({\text{SV}}_{\text{max}}-{\text{SV}}_{\text{min}})/{\text{SV}}_{\text{mean}}\\ \text{SVV}\left(\%\right)=(\chi \times \sigma {\text{AP}}_{\text{max}}-\chi \times \sigma {\text{AP}}_{\text{min}})/\chi \times \sigma {\text{AP}}_{\text{mean}}\\ \text{SVV}\left(\%\right)=(\sigma {\text{AP}}_{\text{max}}-\sigma {\text{AP}}_{\text{min}})/\sigma {\text{AP}}_{\text{mean}}, \end{array}$$

where σAP_max _and σAP_min _are the maximum and minimum SD of arterial pressure during a single respiratory cycle, respectively, and σAP_mean _is the mean SD of arterial pressure over a 20-second interval. Accordingly, the SVV calculation is not influenced by χ, and hence SVV is the respiratory variation of σAP. This means that the entire effect on SVV is based on the variation in the SD of arterial pressure, which should track respiratory changes in left ventricular SV closely [15].

After zeroing the system against atmosphere, the arterial waveform signal fidelity was carefully checked using a fast flush test. A stable hemodynamic condition with no damping of the arterial pressure waveform was a prerequisite for hemodynamic measurements. CO, SV and SVV values were obtained and averaged as the means of three consecutive measurements. Cardiac power output (CPO), a measure of the hydraulic efficiency of the heart, was calculated as (CO × MAP)/451 [16].

### Arterial pressure measurements and arterial tone parameters

The arterial pressure signal was recorded from the bedside monitor connected to the FloTrac pressure transducer. MAP was determined by planimetry, and the trend was recorded every 10 seconds during the same 1-minute period for Vigileo-derived parameters and arterial pressure waveform recordings. The mean of six consecutive measurements for MAP, SAP, diastolic pressure (DAP) and arterial pulse pressure (PP) was used for statistical purposes.

Ea_dyn _was computed as the PPV/SVV ratio. SVR was calculated as SVR = (MAP - central venous pressure (CVP)) × 80/CO. The ratio of pulse pressure (SAP - DAP) to stroke volume (PP/SV) was also calculated as a crude measure of arterial stiffness [17-19]. Although this index has demonstrated underestimate the total arterial stiffness measured by others methods [20,21], since assumes that the total stroke volume is buffered in the elastic arteries during systole without any peripheral outflow, it has been proved to be useful for estimating and detecting changes in arterial stiffness clinically [17,22].

### Study protocol

All the patients were ventilated in supine position in controlled-volume mode with the Puritan Bennett 840 (Tyco Healthcare, Mansfield, MA, USA) or Servo i (Maquet, Bridgewater, NJ, USA) ventilators and temporally paralyzed (0.1 mg/kg vecuronium bromide) if spontaneous inspiratory efforts were detected on the airway pressure curve displayed on the respiratory monitor. During data collection, supportive therapies, ventilatory settings and vasopressor therapy were kept unchanged. A set of hemodynamic measurements was obtained at baseline and after volume expansion (VE), consisting of 500 mL of synthetic colloid (Voluven 6% hydroxyethyl starch; Fresenius Kabi, Bad Homburg, Germany) administered over 30 minutes via an infusion pump.

### Statistical analysis

Normal distribution of data was tested using the D'Agostino-Pearson test for normality. The results are expressed as means ± SD unless otherwise indicated. Patients were classified according to the MAP increase after VE in MAP responders (≥15%) and MAP nonresponders (<15%), respectively. This threshold was selected assuming a perfect arterial pressure-flow coupling of 1:1 and optimal mechanical efficiency, so an increase of 15% in SV should increase MAP by 15% [6,23]. Differences between MAP responders and MAP nonresponder patients were compared by means of an independent sample *t*-test and by the Mann-Whitney *U *test for non-normally distributed variables. The effects of VE on hemodynamic parameters were assessed using a paired Student's *t*-test and the Wilcoxon rank-sum test for non-Gaussian data. Comparisons for categorical variables were performed using the χ^2 ^test. The relationships between variables were analyzed using a linear regression method. Multiple regression analysis was used to study the contribution of each arterial tone parameter with arterial pressure changes after VE. The area under the receiver-operating characteristic (ROC) curves for Ea_dyn_, PP/SV ratio, baseline MAP and SVR according to MAP response to fluid administration were calculated and compared using the Hanley-McNeil test. ROC curves are presented as area ± SE (95% confidence interval). *P *< 0.05 was considered statistically significant. Statistical analysis was performed using MedCalc for Windows version 11.3.3.0 (MedCalc Software bvba, Mariakerke, Belgium).

## Results

### Patients

Twenty-six patients were initially eligible for the study, but one patient was excluded from analysis because SV did not increase ≥15% after VE. The main characteristics of the studied population are summarized in Table 1. The use of vasopressor therapy did not differ between MAP responder and MAP nonresponder patients. Neither tidal volume, nor respiratory rate, nor inspired oxygen fraction nor positive end-expiratory pressure was significantly different between MAP responders and MAP nonresponders. Volume administration was performed mostly because of the presence of the combination of hypotension and oliguria (84%).

**Table 1:** Characteristics and demographic data of study population (*n *= 25)^a^

Parameter	Value
Age (yr)	61 ± 13
Gender (M/F)	15/10
Weight (kg)	75.3 ± 15.3
Height (cm)	168.7 ± 7.8
Body surface area (m^2^)	1.87 ± 0.19
Body mass index (kg m^-2^)	24.5 ± 5.8
APACHE II score at admission	17.7 ± 5.8
Plasma lactate level, mM/L	2.16 (1.25 to 4.1)
Death, *n *(%)	11 (44)
ICU stay before inclusion (days)	1 (1 to 2)
Ventilator settings	
Tidal volume, mL/kg ideal body weight	8.6 ± 1.2
Respiratory rate, breaths/min	18.5 (17 to 20)
Total PEEP, cm H_2_O	7.9 ± 5.9
FiO_2_, %	74 ± 19.6
SaO_2_, %	98.5 (95 to 99)
Vasoactive agents, *n *(dose in μg kg^-1 ^min^-1^)	
Norepinephrine	12; 0.69 ± 0.39
Dobutamine	3; 7.59 ± 1.31
Analgesic and sedative drugs	
Morphine, *n *(dose in mg h^-1^)	3; 4 (3.25 to 4.75)
Fentanyl, *n *(dose in μg kg^-1 ^h^-1^)	8; 1.81 ± 0.6
Remifentanyl, *n *(dose in μg kg^-1 ^min^-1^)	11; 0.13 ± 0.07
Midazolam, *n *(dose in mg kg^-1 ^h^-1^)	14; 0.12 ± 0.05
Acute circulatory failure origin, *n *(%)	
Sepsis	
Abdominal	11 (44)
Pulmonary	3 (12)
Hemorrhagic shock	4 (16)
Postoperative	4 (16)
Others	3 (12)

^a^Values are expressed as means ± standard deviations, medians expressed as the 25^th ^to 75^th ^percentile or absolute numbers as appropriate. APACHE II, Acute Physiologic and Chronic Health Evaluation; ICU, intensive care unit; PEEP, positive end-expiratory pressure; FiO_2_: inspired oxygen fraction; SaO_2_, arterial oxygen saturation.

### Hemodynamic response to volume expansion

**Figure 1:**
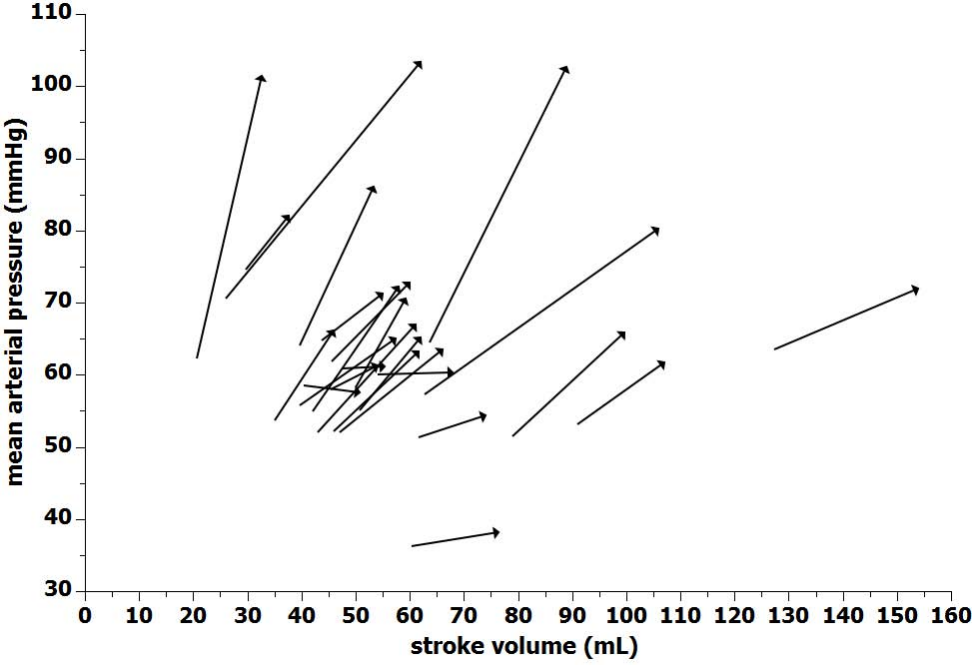
**Mean arterial pressure (MAP) and stroke volume response to volume loading**. Arrows indicate individual changes in stroke volume and mean arterial pressure after fluid administration.

The effects of VE on hemodynamic parameters are summarized in Table 2. In the whole population, VE was associated with a percentage gain in CO of 18.66% (12.16% to 28.61%; *P *< 0.0001), from 5.18 ± 1.73 L/min to 6.25 ± 1.75 L/min; a percentage gain in SV of 26.96% (21.99% to 39.99%; *P *< 0.0001), from 46 mL (40.17 mL to 60.66 mL) to 61 mL (54.75 mL to 74.58 mL); a percentage gain in MAP of 21.5% ± 17.1% (*P *< 0.0001), from 57.86 ± 7.56 mmHg to 70.59 ± 15.27 mmHg; a percentage gain in CPO of 36.36% (24.3% to 63.19%; *P *< 0.0001), from 0.66 ± 0.22 W to 0.96 ± 0.3 W; and an increase in CVP from 7.3 ± 4 mmHg to 10.4 ± 3.8 mmHg (*P *< 0.0001). Overall systemic vascular resistance did not change after VE. Fluid administration induced a ≥15% increase in MAP in 16 patients (MAP responders). Individual changes in SV and MAP after fluid administration are represented in Figure 1. The VE-induced increase in SV was correlated with an increase in MAP (*r*^2 ^= 0.37; *P *= 0.001), SAP (*r*^2 ^= 0.50; *P *= 0.0001), DAP (*r*^2 ^= 0.22; *P *< 0.05) and PP (*r*^2 ^= 0.79; *P *< 0.0001).

**Table 2:** Effects of volume expansion in hemodynamic parameters on responder patients (mean arterial pressure increase ≥15%) and nonresponder patients (*n *= 25)^a^

Parameter	Preinfusion	Postinfusion
CO, L/min		
Responders	5.06 ± 1.64	6.26 ± 1.35^d^
Nonresponders	5.38 ± 1.94	6.23 ± 2.40^c^
HR, beats/min		
Responders	107.12 ± 22.73	96.15 ± 23.97^c^
Nonresponders	99.52 ± 23.65	93.56 ± 24.11^c^
SV, mL		
Responders	48.85 ± 18.04	67.56 ± 21.39^e^
Nonresponders	56.68 ± 28.34	69.44 ± 33.96^d^
MAP, mmHg		
Responders	57.41 ± 5.66	75.41 ± 14.88^e,f^
Nonresponders	58.65 ± 10.49	62.01 ± 12.46^b^
SAP, mmHg		
Responders	82.43 ± 11.36	112.28 ± 19.99^e,g^
Nonresponders	83.9 ± 10.96	89.47 ± 14.82
DAP, mmHg		
Responders	45.68 ± 7.67	55.43 ± 14.01^d^
Nonresponders	45.6 ± 10.09	46.14 ± 11.72
PP, mmHg		
Responders	36.76 ± 14.63	56.82 ± 17.8^e^
Nonresponders	38.29 ± 11.51	43.22 ± 13.49
CVP, mmHg		
Responders	7.13 ± 4.73	10.35 ± 4.69^e^
Nonresponders	7.33 ± 2.9	10.41 ± 2.23^c^
CPO, W		
Responders	0.64 ± 0.22	1.04 ± 0.27^e^
Nonresponders	0.69 ± 0.25	0.84 ± 0.33^c^
PPV, %		
Responders	25.26 ± 9.89^f^	9.38 ± 4.45^e^
Nonresponders	15.07 ± 6.56	6.81 ± 4.47^c^
SVV, %		
Responders	19.14 ± 6.25	10.87 ± 4.55^e^
Nonresponders	19.78 ± 7.59	10.52 ± 5.39^c^

^a^Data are expressed as means ± standard deviations; CO, cardiac output; HR, heart rate; SV, stroke volume; MAP, mean arterial pressure; SAP, systolic arterial pressure; DAP, diastolic arterial pressure; PP, arterial pulse pressure (diastolic pressure minus systolic pressure); CVP, central venous pressure; CPO, cardiac power output (mean arterial pressure × cardiac output/451); PPV, pulse pressure variation; SVV, stroke volume variation; ^b^*P *< 0.05; ^c^*P *< 0.01; ^d^*P *< 0.001; ^e^*P *< 0.0001, postinfusion vs. preinfusion; ^f^*P *< 0.05; ^g^*P *< 0.01 responders (mean arterial pressure increase ≥15% after volume expansion) vs. nonresponders.

### Effects of VE on arterial tone parameters

**Figure 2:**
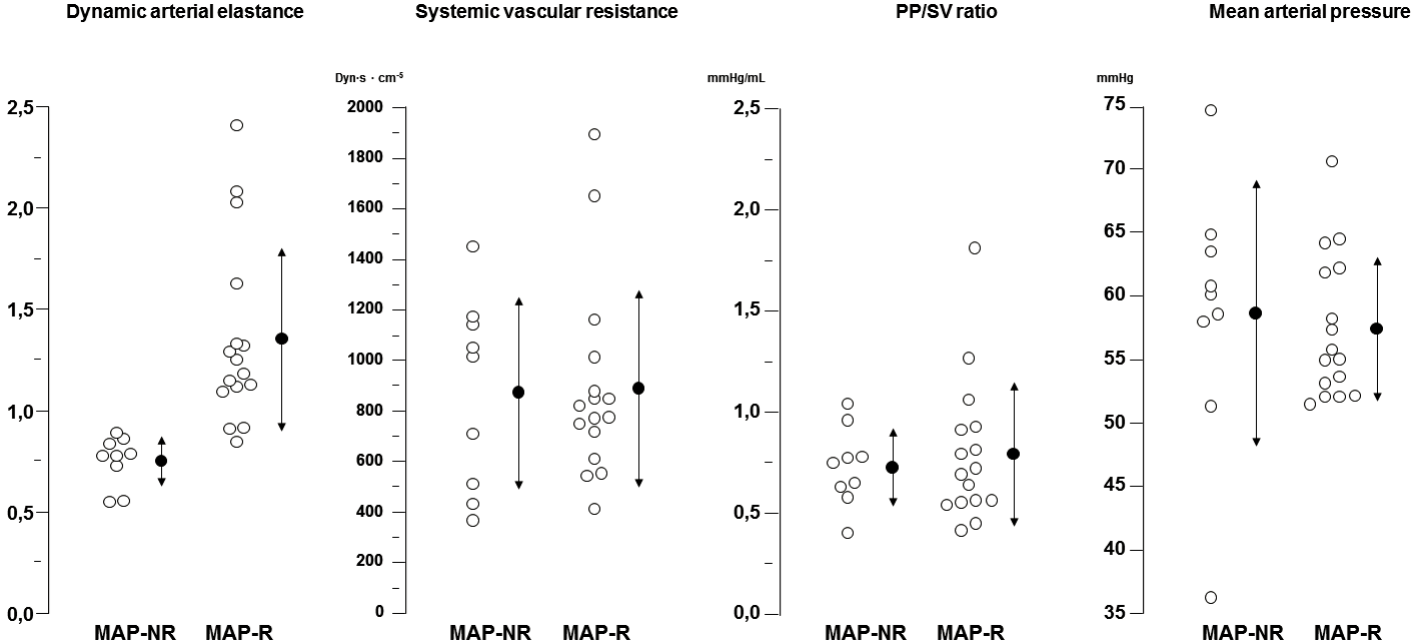
**Distribution of individual arterial tone parameters at baseline**. Individual values (open circles) and mean ± SD (closed circles) of arterial tone parameters before fluid administration in MAP responders (MAP-R) and MAP nonresponders (MAP-NR) with regard to dynamic arterial elastance (Ea_dyn_), systemic vascular resistance (SVR), pulse pressure to stroke volume ratio (PP/SV ratio) and baseline MAP.

The effects of VE on arterial tone parameters are displayed in Table 3. Individual values are shown in Figure 2. At baseline, only Ea_dyn _was significantly different between MAP responders and MAP nonresponders. In the MAP responder group, fluid loading was also associated with a significant decrease in Ea_dyn _by 49.1% ± 38.3%. There was no relationship between Ea_dyn _and the other arterial tone parameters.

**Table 3:** Effects of volume expansion on arterial tone parameters on responder patients (mean arterial pressure increase ≥15%) and nonresponder patients (*n *= 25)^a^

Parameter	Preinfusion	Postinfusion
Dynamic arterial elastance		
Responders	1.34 ± 0.45^c^	0.85 ± 0.21^b,e^
Nonresponders	0.75 ± 0.12	0.64 ± 0.21
SVR, dyn s cm^-5^		
Responders	889.66 ± 392.03	881.19 ± 344.16
Nonresponders	870.95 ± 379.38	774.42 ± 377.17^d^
PP/SV, mmHg/mL		
Responders	0.79 ± 0.36	0.90 ± 0.37^b,d^
Nonresponders	0.73 ± 0.19	0.66 ± 0.16

^a^Data are expressed as means ± standard deviations; SVR, systemic vascular resistance; PP, pulse pressure (systolic minus diastolic pressure); SV, stroke volume; ^b^*P *< 0.05, ^c^*P *< 0.0001 responders (mean arterial pressure increase ≥15% after volume expansion) vs. nonresponders; ^d^*P *< 0.05, ^e^*P *< 0.0001 postinfusion vs. preinfusion.

**Figure 3:**
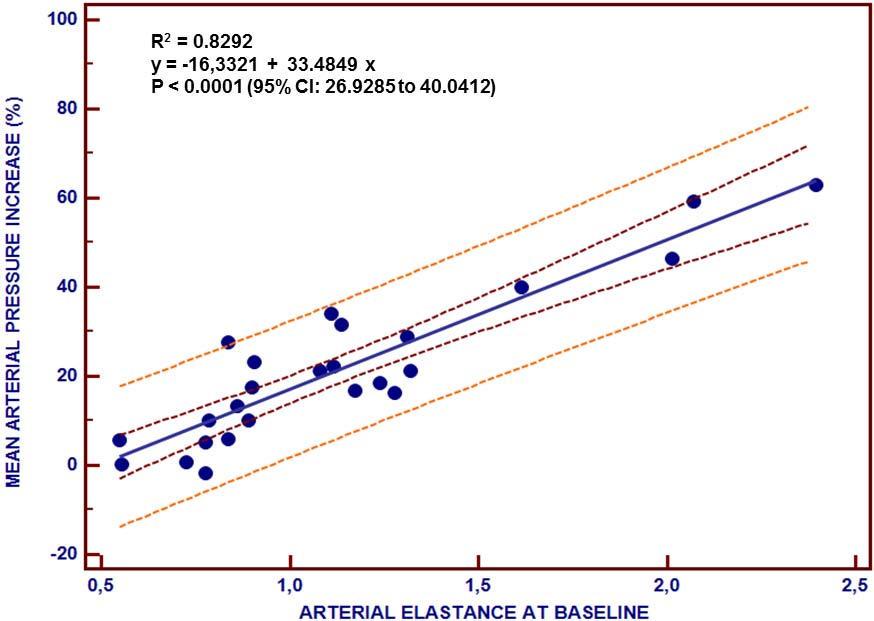
**Dynamic arterial elastance and mean arterial pressure change relationship**. Linear regression analysis of the relationship between baseline dynamic arterial elastance and changes in mean arterial pressure after volume administration are shown. Dashed lines represent 95% confidence intervals for the regression line (solid line).

Before volume administration, Ea_dyn _was correlated with VE-induced changes in MAP (*r*^2 ^= 0.83; *P *< 0.0001), SAP (*r*^2 ^= 0.66; *P *< 0.0001), DAP (*r*^2 ^= 0.81; *P *< 0.0001) and PP (*r*^2 ^= 0.40; *P *< 0.001) (Figure 3). In contrast, none of the other studied arterial tone parameters were related to changes in arterial pressure produced by VE. Fluid-induced decrease in Ea_dyn _was also correlated with changes after volume administration in MAP (*r*^2 ^= 0.78; *P *< 0.0001), SAP (*r*^2 ^= 0.70; *P *< 0.0001), DAP (*r*^2 ^= 0.75; *P *< 0.0001) and PP (*r*^2 ^= 0.40; *P *< 0.001).

### Prediction of arterial pressure response to volume administration

**Figure 4:**
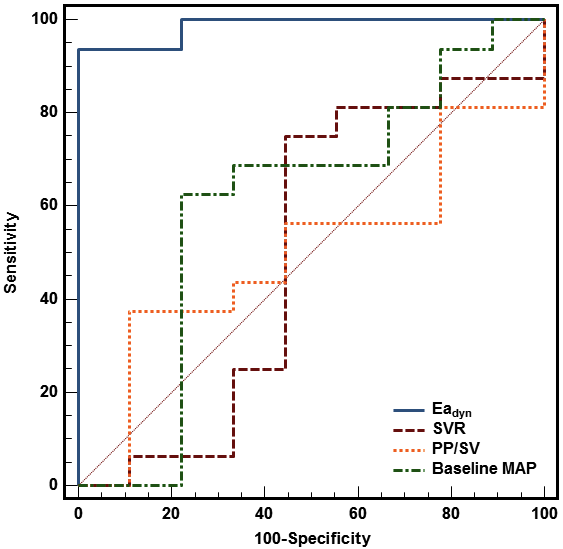
**Comparison of receiver operating characteristics curves regarding the ability of studied arterial tone parameters to discriminate MAP responder patients (MAP increase ≥15%) and MAP nonresponder patients after volume expansion**. Dynamic arterial elastance (Ea_dyn_), 0.986 ± 0.02; systemic vascular resistance (SVR), 0.503 ± 0.12; baseline mean arterial pressure, 0.604 ± 0.12; ratio of pulse pressure to stroke volume (PP/SV), 0.50 ± 0.12. All values are means ± SD.

The area under the ROC curve for the prediction of VE on MAP for Ea_dyn _at baseline (0.986 ± 0.02; 95% CI, 0.84-1) was significantly higher than the areas under the ROC curve for SVR (0.503 ± 0.12; 95% CI, 0.3-0.71; *P *= 0.0001), baseline MAP (0.604 ± 0.12; 95% CI, 0.39-0.79; *P *< 0.001) and PP/SV (0.50 ± 0.12; 95% CI, 0.3-0.7; *P *= 0.0001) (Figure 4). A baseline Ea_dyn _value >0.89 predicted an increase of ≥15% in MAP after fluid administration with a sensitivity of 93.75% (95% CI, 69.8%-99.8%) and a specificity of 100% (95% CI, 66.4%-100%), a positive predictive value of 100 (95% CI, 78.2%-100%) and a negative predictive value of 90 (95% CI, 55.5-99.7%).

## Discussion

The main finding of this study is that Ea_dyn_, defined as the PPV/SVV ratio, efficiently predicted the arterial pressure response to fluid loading in hypotensive, preload-dependent patients with acute circulatory failure.

Since initial hemodynamic resuscitation should be targeted to achieve not only adequate CO but also adequate MAP to guarantee perfusion pressure to all vascular beds [9,10], determining whether a patient is preload-dependent only provides half of the answer, because the arterial pressure response to volume administration depends on arterial tone. Thus, for a given SV increase, the greater the arterial tone, the greater the expected boost in arterial pressure [8].

In our study, only 64% of the hypotensive, preload-dependent patients increased MAP after fluid administration. Neither peripheral vascular resistance, nor the PP/SV ratio, nor the degree of hypotension, defined by the baseline MAP, predicted a subsequent increase in arterial pressure.

Although SVR has traditionally been used to characterize overall arterial tone, this parameter represents primarily the vascular smooth muscle tone at the level of small arteries and arterioles, where a complex system of neurohormonal and local factors adjusts the vessel caliber to protect the capillaries from changes in pressure and to keep capillary perfusion pressure constant [3]. As SVR is not homogeneously distributed along the arterial vascular tree and essentially provides a quantification of arteriolar vasomotor activity, it has been considered an inappropriate and incomplete assessment of arterial tone [24]. Not surprisingly, in our study population, fluid administration did not affect SVR in spite of changes in arterial pressure. Moreover, in MAP responder patients, preinfusion SVR did not correlate with volume-induced increases in arterial pressure or changes after fluid administration, suggesting that arterial pressure changes in these patients were not related to arteriolar vasomotor modulation.

Because arterial pressure results from the phasic interaction of blood ejected from the left ventricle and the arterial system, the pulsatile pressure-flow relationship has been used to describe arterial input impedance [4-6]. This relation provides a more comprehensive description of the arterial load faced by the ejecting ventricle, since it incorporates other components of the arterial system, including total arterial compliance, characteristic impedance or the effects of arterial wave reflections, as well as the ratio of mean pressure to mean flow [3,7,25]. Since the evaluation of arterial input impedance requires measuring pressure and flow waves and the application of complex Fourier analysis, the ratio of PP to SV has been proposed as a simple and gross estimation of the pulsatile component of the arterial system and a surrogate measure of the systemic arterial stiffness in clinical practice [17,22,26]. However, in the same way that knowing the values of cardiac preload and CO does not allow the prediction of the response to a fluid challenge, since the response will depend on the slope of the cardiac function curve, the steady-state relationship between pulsatile pressure and pulsatile flow, as measured by the PP/SV ratio, is theoretically variable with different states of arterial tone and influenced by factors such as aging and pathologies such as arterial hypertension [19,27,28]. Therefore, for the same static pressure-flow relationship, the VE-induced increase in arterial pressure should depend on arterial tone; thus, as our results show, prediction of the arterial pressure response by PP/SV should be infeasible [8].

On the contrary, as Pinsky has pointed out, Ea_dyn _represents neither a steady nor pulsatile component of arterial system, but instead a functional measure of central arterial tone [1,8]. During mechanical ventilation, swings in intrathoracic pressure induce cyclic changes in left ventricular SV by intermittently varying right ventricular preload. The magnitude of these changes defines the degree of preload dependence of a patient and the position on the Frank-Starling curve, and these changes have been widely used as indicators of fluid responsiveness [12]. Thus, simultaneous measurements of arterial pulse pressure and left ventricular SV during passive mechanical ventilation should provide an actual assessment of the pressure-volume relationship and an accurate measurement of arterial tone. Ea_dyn_, therefore, rather than absolute values of pressure and flow, depicts the actual slope of the pressure-volume relationship using the cyclic changes in left ventricular SV during a single mechanical respiratory cycle. So, Ea_dyn _should be interpreted as a functional approach to arterial tone assessment in the same way that preload responsiveness parameters attempt to predict the hemodynamic response to a change in cardiac preload.

According to our results, a patient with an Ea_dyn _value <0.89 will not have an increase MAP with volume administration, which pragmatically means that vasopressors should be added along with fluids to increase the patient's CO and MAP. By contrast, an Ea_dyn _value >0.89 indicates that fluid loading alone will significantly raise blood pressure, and thus the use of vasoactive drugs can be delayed. These results are in accord with a previous algorithm proposed by Pinsky as part of a functional management protocol based on ventriculoarterial coupling [1,8]. According to this algorithm, if a balanced system should present an Ea_dyn _close to 1 and changes >20% reflect real variations in arterial elastance, then the normal value for the PPV/SVV ratio should be between 0.8 and 1.2. In our study, Ea_dyn _measurement does not represent an online monitoring method (since PPV value was obtained from a *post hoc *offline analysis); however, with the current technology available, it might be possible to easily obtain both parameters simultaneously, allowing continuous assessment of Ea_dyn _at the bedside.

Interestingly, from a theoretical point of view, the evaluation of Ea_dyn _should not necessarily be limited by some of the restrictions imposed on the fluid responsiveness parameters. In particular, the assessment of arterial tone by Ea_dyn _could be used in spontaneously breathing patients or in patients with low tidal ventilation, since the relation between PPV and SVV should still be valid under these circumstances [5]. Furthermore, another potential advantage of the combined evaluation of the preload dependence and arterial tone by Ea_dyn _could be the prediction of the expected increase in hydraulic efficiency measured by the cardiac power output. Hypothetically, for the same fluid responsiveness degree, a preload-dependent patient with a higher Ea_dyn _value would respond with a more marked increase in MAP, higher CPO, and thus a better improvement in the mechanical efficiency of hydraulic power transfer from the left ventricle to the peripheral circulation [1]. These assumptions, although physiologically reasonable, require confirmation by further studies.

Some important limitations of our study should be addressed. First, the SVV value was obtained not from the actual arterial blood flow, but from the results of arterial pressure analysis using the Vigileo hemodynamic monitor. Pinsky has already warned against the use of pulse contour-derived SVV to track rapid changes in SV, as occurs during a single mechanical breath [29,30]. In this regard, Vigileo-derived SVV has been confirmed as a valuable predictor of fluid responsiveness [11,31] and equivalent to SVV measured by transthoracic echocardiography [15]. However, since SVV is actually the respiratory variation of SD of arterial pressure, as the χ factor is updated only every minute, the possibility of a mathematical coupling cannot be excluded. Also, this study was targeted to a specific group of patients with a preserved preload dependence condition and manifest arterial hypotension, so that extrapolation of our results to other situations should be considered with caution. However, the assessment of arterial tone by Ea_dyn _clearly responds to a concrete, often stressful situation with which clinicians must habitually deal in their daily practice. Finally, hemodynamic resuscitation should be aimed not only at restoring blood flow and perfusion pressure but also at maintaining adequate tissue oxygenation. Increasing MAP to a predefined level does not guarantee sufficient oxygenation to all tissues nor can be generalized to all patients [31]. Furthermore, systemic hypotension is not always present in shock, and restoration of normal arterial blood pressure does not exclude maldistribution of blood flow to vital organs. However, it seems reasonable that an acceptable minimum level of MAP is necessary to avoid further hypoperfusion [10].

## Conclusions

In conclusion, in our study, the functional assessment of arterial tone by the Ea_dyn_, defined as the PPV/SVV ratio, predicted the arterial pressure response to volume loading in hypotensive, preload-dependent patients with acute circulatory failure. However, because of the small sample size, the specific population studied and the methodological limitations, further validation is required before the application of Ea_dyn _in clinical practice can be recommended.

## Key messages

• Ea_dyn_, defined as the PPV/SVV ratio, accurately predicts the arterial pressure response after volume administration in hypotensive, preload-dependent patients with acute circulatory failure.

• An Ea_dyn _threshold of 0.89 discriminated which patients had increased arterial pressure with fluid administration with a sensitivity of 94% and a specificity of 100%.

• From a practical point of view, patients with an Ea_dyn _value <0.89 require vasopressors along with fluids to increase MAP, whereas patients with an Ea_dyn _value ≥0.89 show an indication that fluid loading alone will increase blood pressure.

## Abbreviations

σPA_max_: maximum standard deviation of arterial pressure during a single respiratory cycle; σPA_mean_: mean standard deviation of arterial pressure over a 20-second interval; σPA_min_: minimum standard deviation of arterial pressure during a single respiratory cycle; CO: cardiac output; CPO: cardiac power output; CVP: central venous pressure; DAP: diastolic arterial pressure; Ea: arterial elastance; Ea_dyn_: dynamic arterial elastance; FiO_2_: inspired oxygen fraction; ICU: intensive care unit; MAP: mean arterial pressure; PEEP: positive end-expiratory pressure; PP: arterial pulse pressure; PP_max_: maximum pulse pressure during a single respiratory cycle; PP_min_: minimum pulse pressure during a single respiratory cycle; PPV: arterial pulse pressure variation; SAP: systolic arterial pressure; SV: stroke volume; SV_max_: maximum stroke volume during a single respiratory cycle; SV_mean_: mean value of SV during 20 seconds for Vigileo monitor; SV_min_: minimum stroke volume during a single respiratory cycle; SVR: systemic vascular resistance; SVV: stroke volume variation; VE: volume expansion.

## Competing interests

MIMG has received consulting fees from Edwards Lifesciences. AGC and MGR declare that they have no competing interests.

## Authors' contributions

MIMG conceived and designed the study, participated in the recruitment of patients, performed the statistical analysis, interpreted the data and drafted the manuscript. AGC participated in the study conception and design, interpreted data and helped draft the manuscript. MGR participated in patient recruitment, data collection, technical support and contributed in the critical review of the manuscript. All of the authors read and approved the final manuscript.
